# Migrant survivors of conflict-related sexual violence accessing a specialist health service in Turin, Italy: a qualitative analysis of clinical forensic interview transcripts

**DOI:** 10.3389/fsoc.2024.1454700

**Published:** 2024-09-09

**Authors:** Elena Rubini, Monica Trentin, Martina Valente, Stefano Cenati, Antonella Canavese, Paola Castagna, Luca Ragazzoni, Sarah Gino

**Affiliations:** ^1^CRIMEDIM—Center for Research and Training in Disaster Medicine, Humanitarian Aid and Global Health, Università del Piemonte Orientale, Novara, Italy; ^2^Department of Translational Medicine, Università del Piemonte Orientale, Novara, Italy; ^3^Department for Sustainable Development and Ecological Transition, Università del Piemonte Orientale, Vercelli, Italy; ^4^Department of Health Sciences, Università del Piemonte Orientale, Novara, Italy; ^5^Emergency Department, Azienda Socio-Sanitaria Territoriale della Valle Olona, Busto Arsizio (Varese), Italy; ^6^Centro Soccorso Violenza Sessuale, Presidio Ospedaliero Sant’Anna, Città della Salute e della Scienza, Torino, Italy; ^7^Department of Surgical Sciences, University of Turin, Torino, Italy; ^8^Direzione Medica dei Presidi Ospedalieri, AOU Maggiore della Carità, Novara, Italy

**Keywords:** conflict-related sexual violence, gender-based violence, sexual violence, conflict, migration

## Abstract

**Introduction:**

The Sexual Violence Relief center *Soccorso Violenza Sessuale* (SVS) is a specialist service, situated in Sant’Anna Hospital, an Obstetrics and Gynecology facility in Turin, North-West Italy. The study aimed to qualitatively analyze the transcripts of interviews routinely conducted by gynecologist and midwife in the first part of the medical examination of migrant patients accessing care at SVS after being subjected to conflict-related sexual violence (CRSV) in their home country or during migration and to explore the adverse outcomes of such violence on their health.

**Methods:**

Interview transcripts were purposely selected to include adult migrant patients (age > 18) subjected to CRSV in the different phases of migration and accessing SVS from January 1st, 2014, to September 4th, 2023. Data was extracted from the SVS archive, anonymized, and thematically analyzed.

**Results and discussion:**

In total, 43 interview transcripts were eligible for inclusion. All of them were related to cisgender women of Sub-Saharan origin describing different forms of violence as a driver for migration. CRSV was disclosed by 18 survivors as occurring in their home country and by 31 in transit (e.g., Libya), the most reported type being rape. 49% of the patients described adverse physical outcomes of CRSV, while 72% reported psychological sequelae. The findings confirm high levels and different modalities of violence throughout the migratory route. Qualitative analysis of interview transcripts served as a valuable source for understanding how survivors described the CRSV they endured, its consequences, as well as other violence encountered during migration.

## 1 Introduction

Conflicts put individuals at risk of different forms of human rights violations, including gender-based violence (GBV), namely “any harmful act perpetrated against a person’s will and based on socially determined gendered roles” (IASC Task Force on Gender and Humanitarian, Assistance, 2005). Conflict-related sexual violence (CRSV) is a specific form of GBV temporally, geographically, or causally connected to a conflict (Preventing Sexual Violence in Conflict Initiative, 2017), a violation of the Sexual and Reproductive Health and Rights (SRHRs) of individuals (Starrs et al., 2018), and a public and global health issue (Heise et al., 1994; Öhman et al., 2020; Sanjel, 2015; WHO, 2002, 2003). GBV, including CRSV, disproportionately affects women and girls (IASC Task Force on Gender and Humanitarian, Assistance, 2005; Pertek and Phillimore, 2022), with 94% of the 2,455 reported cases of CRSV in 2022 affecting these groups (UN, 2022).

The concept of “continuum of violence” traces back to the work of Kelly, who used this expression to highlight the interconnections occurring between different forms of violence and warned against making a distinction between criminal acts and “minor” episodes of GBV (Kelly, 1988). It has been employed also to describe the continuity of violence between the private and public sphere (Gray, 2019; Swaine, 2015) as well as that occurring in times of peace and of conflict (Cockburn, 2004).

For these reasons, conflicts as well as the different forms of violence perpetrated against civilians and other actors should not be understood as punctual events, but rather as a continuum of violence, extending in space and time through flight and displacement (Ferris, 1990; Krause, 2015). Violence affecting migrants must be understood in its structural dimension as deriving from the inequalities and discrimination faced by individuals based on different and intersecting identities (e.g., related to gender, race, ethnic origin, socioeconomic status, legal status), which are instrumentalized against migrants by different institutional actors directly or by failure to protect them from abuse or to support them after its occurrence (Crenshaw, 1989; Davies and True, 2015; Nixon, 2019; Simon-Butler and Mcsherry, 2019; Tastsoglou et al., 2021; Trentin et al., 2024). Research focusing on specific types of GBV in conflict, including CRSV, should equally describe other co-occurring types of abuse, to avoid a hierarchization of violence as well as to understand the cumulative effect of various forms of abuse (Menjívar and Perreira, 2019; Swaine, 2015). Moreover, in contrast to research that solely focused on CRSV used as a weapon of war (e.g., perpetrated by armed actors in a conflict-affected home country), literature is now shifting its attention to other kinds of perpetrators (e.g., smugglers, police, paramilitaries) and geographical locations (e.g., transit countries; Hourani et al., 2021; IOM, 2019; Reques et al., 2020). This encompasses the emerging research focusing on migrants affected by conflict in the different phases of their migratory journey and describing the various forms of violence, including GBV, they experience (Borges, 2024; Hourani et al., 2021; Phillimore et al., 2023; Rubini et al., 2023; Tan and Kuschminder, 2022). As for what concerns migrant women in transit, sexual violence and other types of GBV have been documented in the literature in the Mediterranean Routes and especially through Libya (Amnesty International, 2020; Malakooti, 2019; Reques et al., 2020), as well as in the route Latin America—Mexico—USA (e.g., Mexican migration corridor; Cleaveland and Kirsch, 2020; Infante et al., 2020; Obinna, 2021; Pries et al., 2024; Soria-Escalante et al., 2022). These can take various forms, from rape, to other types of sexual violence, transactional sex, sexual trafficking, up to the point of femicide (Soria-Escalante et al., 2022).

Conflict, violence, GBV, and CRSV can act as drivers for resettling or can occur or be reiterated in the different phases of the migratory route (Morgen, 2013; Pertek and Phillimore, 2022; Pries et al., 2024; WHO, 2022). CRSV provokes negative consequences on the physical, psychological, and social dimensions of health of victims (Rubini et al., 2023; Starrs et al., 2018; WHO, 2002), which may be exacerbated by delayed access to care due to the weakening or the disruption of health systems occurring in conflict-affected settings; for some migrant survivors access to care may occur after a longer time span in transit or host countries, often hindered by restrictive health policies (Acquadro-Pacera, 2024; Castagna et al., 2018; Footer and Rubenstein, 2013; IASC Task Force on Gender and Humanitarian, Assistance, 2005; IOM, 2006; Spitzer et al., 2019). Moreover, the exposure to abuse and violence in the different phases of migration, the possibility to use legal or illegal routes, as well as their safety, equally influence migrants’ health (IOM, 2006).

The Council of Europe Convention on preventing and combating violence against women and domestic violence (e.g., Istanbul Convention) establishes that cases of GBV should be managed by general [e.g., Emergency Departments (EDs)] and specialist services (Council of Europe, 2018, 2011a, 2011b). Specialist support services aim to ensure the empowerment of survivors through allocation of high-quality support and assistance through different forms of provision (e.g., shelters, telephone helplines, counselling centers, support for victims of sexual violence; Council of Europe, 2018, 2011a, 2011b). Among the latter are sexual violence referral or relief centers, which can be managed by non-governmental organizations (NGOs; e.g., anti-violence centers) or by government authorities (Council of Europe, 2018). Anti-violence centers operate with a multi-agency perspective by providing support to survivors of GBV, including referral to the ED.

In Italy, the Ministry of Health adopted in 2017 guidelines for the promotion of an integrated and multidisciplinary approach (e.g., socio-sanitary, legal, and cultural mediation needs) in the management of refugees or individuals with subsidiary protection victims of torture, rape, or other forms of physical, psychological, or sexual violence, also highlighting the role of the medico-legal report in documenting the abuses they endured (Ministero della Salute, 2017).

In Italy, the *Soccorso Violenza Sessuale* (SVS) at Sant’Anna Hospital in Turin and *Soccorso Violenza Sessuale e Domestica* (SVSeD) at Mangiagalli Clinic in Milan, are both situated in two public hospitals in the North-West region. In particular, SVS created a dedicated healthcare pathway for migrant survivors of GBV, including CRSV, regardless of their legal status. Italy is a transit and destination country for migrants, and, even though no exact figures are available, we can presume by using their home countries as a proxy that conflict constitute the reason for fleeing for many of them (Fondazione ISMU, 2023). In specialist centers, the transcript of patients’ interviews on their history of violence, which constitutes the first phase of the medical examination, consists of a detailed report, useful for the clinical and forensic management of cases. To our knowledge, the two specialist centers only published quantitative studies focusing on epidemiological and clinical findings retrieved from databases and medical records concerning the top-to-toe and gynecological examination (Castagna et al., 2018; Gino et al., 2017; Margherita et al., 2021; Simonaggio et al., 2024). Even though this data allows us to understand the epidemiological characteristics of their pool of users, subjective elements emerging through patients’ interviews, which are crucial for health providers to contextualize the episodes of violence suffered by victims of GBV, have never been investigated.

Our research team previously conducted a systematic literature review analyzing qualitative peer-reviewed evidence of CRSV, with an inclusive operational definition encompassing also abuses occurring during migration, as well as describing other types of co-occurring violence (Rubini et al., 2023). We also conducted another systematic review, on the health sequelae of CRSV documented during forensic medical examination, which showed that studies on this matter are generally conducted in high income destination countries (Rubini et al., 2024). In this study we relied on original data to investigate the health outcomes of CRSV, thus complementing the findings of our previous secondary data analyses.

The aims of this study are to qualitatively examine episodes of CRSV endured by migrant women as disclosed by them during admission at SVS of Sant’Anna Hospital in Turin, North-West Italy, including the adverse outcomes on their health, and to investigate how CRSV affected women over the different phases of migration.

The research was guided by the questions “How do migrant women survivors of CRSV describe their history of violence during admission at the SVS of Sant’Anna Hospital in Turin?” and “How did CRSV affect them during the different phases of migration?.” Giving an answer to these questions will contribute to understanding how episodes of CRSV are depicted by victims at access to care and will serve to document what kind of CRSV was endured by migrant women patients of SVS in their home and transit countries.

## 2 Methods

A retrospective qualitative analysis of the transcripts of interviews conducted prior to medical examination was performed to capture how victims of CRSV reported their history of assault at the time of accessing care at SVS and the negative consequences of such violence described by them.

### 2.1 Study setting

In the metropolitan area of Turin, North-West Italy, half of the migrant population among those who have a permit to stay is female (Ministero del lavoro e delle politiche sociali, 2021). The most recent data available showed that among those who have this permit, 11.3% are under some form of protection (Ministero del lavoro e delle politiche sociali, 2021). Namely, 51.5% of them are refugees or asylum seekers, 32.7% have subsidiary protection, and 15.7% have humanitarian protection (Ministero del lavoro e delle politiche sociali, 2021).

Since May 2003, SVS is one of the two specialized centers in Italy offering free assistance, 24/7, to women of 14 years of age or older subjected to different forms of GBV. The service is situated in the ED of Sant’Anna Hospital, an Obstetrics and Gynecology facility in Turin. It provides clinical, forensic, psychological, legal, and social support to survivors in the immediate aftermath of abuse and in the longer period, with a 3 months follow up after the first admission. Gynecologists, obstetric nurses, psychologists, cultural mediators, and social assistants manage in a multidisciplinary perspective the needs of survivors. Access to SVS can occur through the ED, scheduled appointment, referral from other facilities in the metropolitan area of Turin, as well as from public or private third sector organizations operating in the same territory (e.g., anti-violence centers, anti-trafficking networks, migrant shelters, and reception centers). Third sector organizations working with migrant women serve as a contact point between them and SVS, thus integrating the management of their needs as migrants and as survivors of GBV. SVS does not operate in direct contact with border services for migrant women victims of violence, but indirectly through the mediation of third sector organizations working in the migrant reception system as well as in other areas (e.g., GBV, sexual trafficking). After collection of the history of assault and informed consent, the top-to-toe examination is conducted, together with the collection of biological material for forensic genetics and toxicology analyses, clinical screening for STI, and pregnancy test. Consultations take 5 h on average. With a patient-centered, as well as a culturally, trauma, and gender sensitive approach, the service cares for and holistically cures about 100 patients per year.

### 2.2 Data collection, screening, and eligibility criteria

This study qualitatively analyzed the transcripts of the interviews of patients accessing SVS routinely conducted by gynecologist and midwife, with the presence of a cultural mediator, in the first part of the medical examination.

At times, details on symptoms and signs connected to psychological sequelae were also described. Text fragments from the medical records were translated for publication purposes from Italian into English and added to the Results section to enhance the understanding of the study findings.

Interview transcripts were purposely selected to include adult migrant patients (age > 18) subjected to non-partner violence (NPV) in their home country or during migration (see *“Operational definitions”*) and who accessed care at SVS from January 1st, 2014, to September 4th, 2023. This initial screening was performed by one of the research team members (AC) who works as a midwife in the facility. To ensure compliance with eligibility criteria, a second screening was performed (ER, MT). Histories of violence that did not describe episodes of sexual violence, and those not mentioning conflict in any of the phases of migration were excluded, together with accounts where it was not possible to understand whether violence was related to conflict or who the perpetrators were. The interviews whose transcripts were considered eligible for inclusion were extracted from the SVS archive, anonymized, and transferred to a Word file for qualitative analysis.

### 2.3 Data analysis

Thematic data analysis was used (Braun and Clarke, 2006). Researchers familiarized with data by actively reading accounts multiple times. A coding table was created (Additional file 1) with the aim to capture information on conflict, survivors’ demographics, CRSV and its consequences, as well as other experiences of violence and GBV, and migration. To ensure coding accuracy and consistency, each code was given an explicit definition and precise instructions. Interview transcripts were transferred on software Atlas.ti [version 23.4.0.29360]. Coding was performed by two investigators (ER, MT) in the months of October and November 2023. The codes were then inductively organized into patterns and themes.

### 2.4 Operational definitions

For the purpose of the present study, CRSV was understood as “temporally, geographically, or causally” related to conflict (Preventing Sexual Violence in Conflict Initiative, 2017; UN Security Council, 2023). Not only CRSV endured in the home country and possibly provoking displacement and episodes of re-victimization was included, but also CRSV in conflict-affected transit countries, also considering individuals who migrate due to non-conflict related reasons.

Conflict encompasses the International Humanitarian Law definitions of international and non-international armed conflict (ICRC, 2008), but also persecution (MSF, n.d.), political violence (Balcells, 2015; Kalyvas, 2001), violence due to cults (EASO, 2021), terrorism (Sinai, 2008; Teichman, 1989), and gang violence (Applebaum and Mawby, n.d.; Rodríguez Serna, 2016) when not contrasted by State authority. As for what concerns Libya, we understand this State in the Maghreb region of North Africa as conflict-affected by civil war in the years 2011–2021, and up to the present day as under the control of different armed groups and militias (Non-international armed conflicts in Libya, 2022; US Department of State, Office to monitor and combat trafficking in persons, n.d.). Data related to Libya was collected when hostilities were directly mentioned or when specific terms were used (e.g., militia men, rebels, men in military uniforms, policemen, detention centers or prisons, Asma Boys).

The terms “survivor” and “victim” will be used interchangeably to refer to individuals subjected to SV; the first term serves to highlight the process of recovery, while the second calls attention to the criminal nature and the severity of acts of GBV (Rubini et al., 2023).

“Migrant” is intended as an umbrella term to refer to individuals who move away from their place of usual residence for a variety of reasons (IOM, n.d.).

### 2.5 Ethics

The study was approved by the Ethics Committee of the AOU Città della Salute e della Scienza di Torino—AO Ordine Mauriziano di Torino (CE 112/2020) and complies with the Declaration of Helsinki for experiments involving humans (2013), to the General Data Protection Regulation (2018), and to the Provision no. 146/2019 of the Italian Privacy Guarantor. For information about consent to participate, please see the Informed Consent Statement.

## 3 Results

In total, 43 interview transcripts were eligible for inclusion. Other 44 were excluded since they did not describe episodes of CRSV. This section will explore the demographic characteristics as well as conflict and migratory experience of survivors, including the reason for fleeing their home country, the CRSV sustained, the adverse outcomes of CRSV on their health, as well as other violence endured (e.g., conflict- and migration-related nonsexual violence and other GBV).

### 3.1 Characteristics of survivors

All 43 patients in our sample were cisgender women of Sub-Saharan origin living in Italy at the time of accessing SVS. When seeking care, their self-reported age ranged from 19 to 48, with the median age being 31. Among them, 20 were mothers, and only 5 fled with their children.

Means of livelihood in home or transit countries were mentioned only by 15 patients. While two survivors were, respectively, a social educator and a nurse, most women had informal jobs. They were housemaids (*n* = 7), street sellers (*n* = 5), hairdressers (*n* = 2), babysitters (*n* = 1), farmers (*n* = 1), dishwashers (*n* = 1), caregivers (*n* = 1), or sex workers (*n* = 1).

### 3.2 Migratory experience

**Figure 1:**
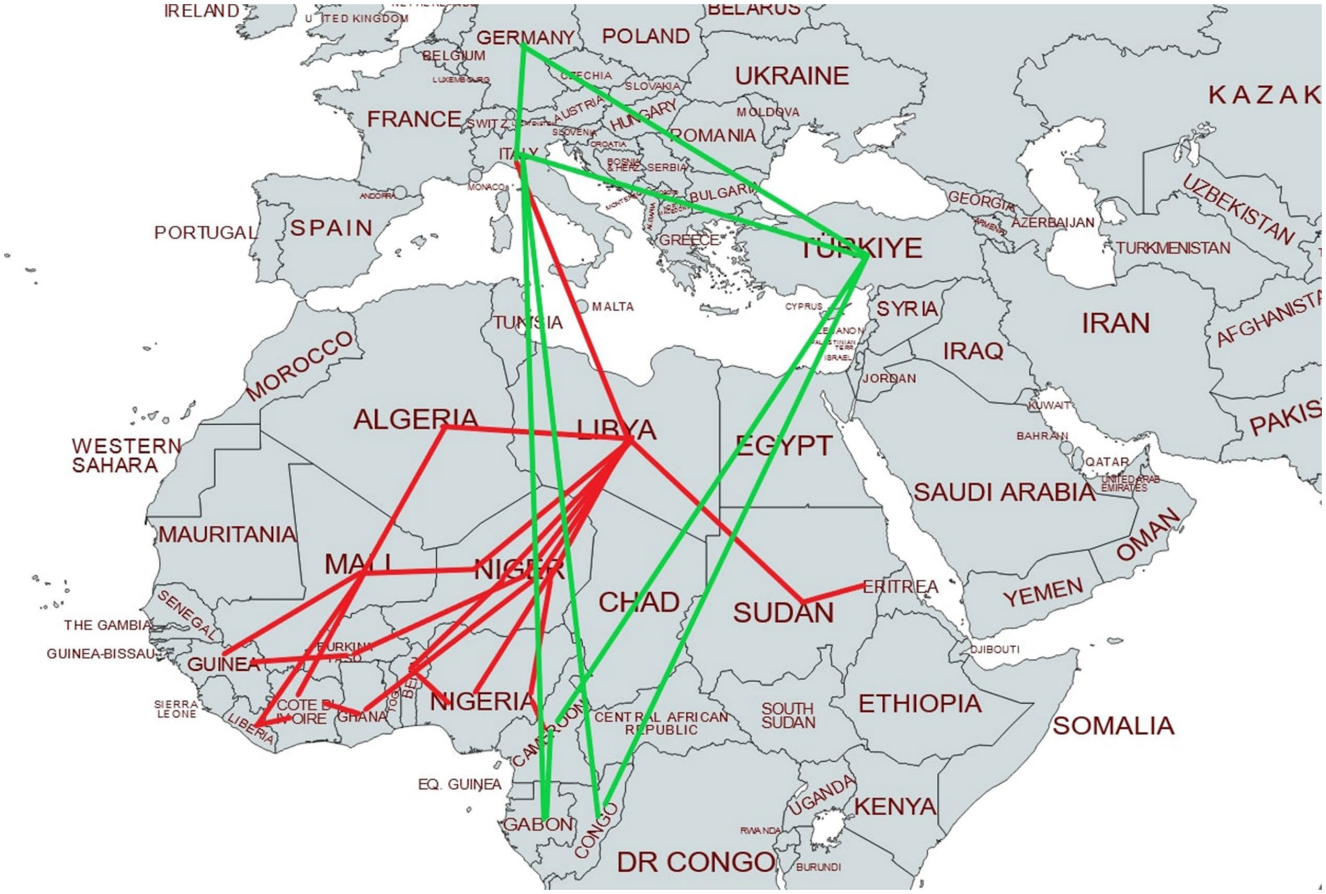
Migratory route of survivors accessing care at SVS. Please note that it represents only the journey of patients who described all phases of migration. A green line represents travel by plane, while a red line stands for other means of transport (e.g., car, bus, taxi, van, and pick up).

For 37 women (86%) Libya was the last disclosed transit country before arrival in Italy. Figure 1 provides a map of the migratory route as described by survivors accessing care at SVS.

When mentioned, migrant women whose last transit country was Libya reported means of transportation used to arrive in Italy were “boats” (*n* = 20) or “rubber boats” (*n* = 7). A small number of survivors described traveling by plane (*n* = 4, 9%) directly from their home country (Congo, *n* = 2), or from transit countries (Gabon and Türkiye, *n* = 1 each).

Information on year of admission at SVS and home country can be found in Table 1 (Additional file 2).

**Table 1:** Characteristics of the pool of users of SVS survivors of CRSV.

Year of admission at SVS	*n*
2014	2
2015	1
2016	29
2017	5
2018	2
2019	1
2022	2
2023	1
**Home country**	**n**
Guinea	1
Ivory Coast	14
Nigeria	14
Congo	5
Cameroun	6
Somalia	1
Burkina Faso	1
Eritrea	1

### 3.3 Conflict in home and transit country

Information on the country where survivors encountered hostilities, type of conflict, and number of survivors who reported it can be found in Table 2. Conflict was described as occurring in their home countries by 25 patients, while 31 encountered hostilities in Libya.

**Table 2:** Table showing the countries where survivors encountered conflict and type.

Country where conflict was mentioned	Home country	Transit country	Type of conflict as reported by survivors	% of survivors (*N* = 43) describing conflict there
Guinea	X		Political opposition	2%
Ivory coast	X		Armed conflict Civil war Political crisis Persecution of specific ethnic groups/tribes Post-electoral crisis Electoral crisis War with bombings and violence targeting civilians Rebels Post-electoral civil war	28%
Congo	X		Political opposition. Clan persecution Political instability	12%
Cameroun	X		Terrorist groups Civil war Political opposition	7%
Somalia	X		Tribal war	2%
Nigeria	X		Eye Confraternity Civil war	5%
Eritrea	X		Political persecution	2%
Libya		X	Rebels Terrorist gangs Militias Civil war	72%

### 3.4 Reason for fleeing

For most women, disclosed causes for migration were connected to different forms of GBV (*n* = 9, 21%) and to conflict (*n* = 26, 60%). In the first case, violence targeted both survivors of CRSV as well as other members of their family (e.g., husband).

Occasionally, migrants described economic (e.g., poverty and lack of economic safety for their children), work-related reasons, and search for better healthcare as strategic drivers for migration.

### 3.5 CRSV

#### 3.5.1 Description of CRSV

During the interview, the history of assault was collected in first (*n* = 28) or third person (*n* = 15). In most cases the history of violence as reported in the medical records was narrated by enumerating the different episodes of violence (CRSV, other violence, and other GBV) endured by survivors in an objective and detached manner. At times, professionals working in SVS described the difficulties patients had in disclosing the violence they suffered. One survivor accessed SVS multiple times since the suffering prevented her from retelling the events. The psychological sequelae emerged through signs’ annotations taken by the gynecologist or midwife.

#### 3.5.2 CRSV in home country

CRSV occurring in the home country was mentioned by 18 survivors (42%), of which 14 described being subjected to co-occurring nonsexual violence (78%).

All of them described being raped, with different modalities (e.g., single episodes and perpetrators, multiple or gang rape, transactional sex). Some disclosed being pregnant at the time of assault, with aggressors attempting to induce miscarriage.

“We were taken hostage for 10 days. I was pregnant. We were subjected to physical violence — beatings with the use of hands, feet, sticks I still have the scars of — and to sexual violence: I was vaginally abused multiple times by different men.” (Cameroun)

Others described policemen trying to force the victim’s brothers to rape her and her sister. SV was sometimes associated with witnessed rape of family members or witnessed instrumentation, and unwanted sexual touching.

Witnesses of episodes of CRSV were family members, including children.

Perpetrators were described as members of armed forces, police officers, Boko Haram members, rebels, individuals who broke into the victim’s home (e.g., “men” or “rebels,” “supporters of a different political party”), and were at times defined in generic terms (e.g., “men”, “a group of men,” “armed men”).

CRSV was reportedly perpetrated in a variety of locations: a stadium, indoors, the perpetrators’ car, terrorist groups’ hideouts or other secluded places, the street, police centers, and in prison. A pattern of CRSV occurring in the survivors’ home emerged (*n* = 8, 44%).

“One day, the rebels broke into our home and beat us. My niece was beaten to death, my sister-in-law and I were raped.” (Ivory Coast)

Reported co-occurring nonsexual violence included use of blunt force (through use of natural weapons, sticks, and belts), grabbing, pushing, hair dragging, ejection from a vehicle, imprisonment, kidnapping and being taken hostage, death threats, including with weapons, torture, being tied with a rope, witnessed killing of family members, and kidnapping of family members.

In particular, one survivor described excruciating physical and sexual violence. After being subjected to CRSV on more than one occasion, she endured 6 years of prison where she was constantly sexually victimized and other 5 years in a different detention facility where she was raped daily. Co-occurring violence encompassed forced labor in inhuman conditions, including being forced to throw dead bodies of other prisoners into a river, beatings with a burning machete, denial of water, care, and of access to sanitation facilities (e.g., showers and toilets). During episodes of CRSV she was tortured and beaten. The survivor expressed living in a state of constant fear:

"They told us we were sentenced to death, and we lived with that terror. I lost any hope, I wanted my life to end, I wanted to die." (Congo)

#### 3.5.3 CRSV in transit country

CRSV in transit countries was reported by 31 survivors (72%), of which 22 described co-occurring nonsexual violence (51%). Overall, six women disclosed victimization both in home and transit countries (14%).

Survivors described being subjected to rape (e.g., single episodes and perpetrators, gang and multiple rape, penile, with fingers, and instrumentation, vaginal, oral, and anal) including while being pregnant, attempted forced abortion, torture-induced miscarriage, forced nudity, and unwanted sexual touching.

A common reported pattern was being kidnapped, scammed, or arrested and sold to brothels (also described as “houses,” “prisons,” or “detention houses”) together with other women, and being brought to other places during the night to be sexually exploited.

“When we arrived at the other house, we understood something was wrong: there were many women. Some men beat and rape us every day. Often a group of men did that from 10 pm until 6 am.” (Ivory Coast)

At times, individuals who paid for victims’ release from detention centers were the ones who sold them to brothels. A survivor described being forced into prostitution while pregnant, with a *“madame”* attempting unwanted pharmacological abortion on her. Another reported being trafficked and sold to a “mentally disturbed Arab man” together with other women, and enduring physical abuse.

Other women victims of violence, the survivors’ husbands, or their children witnessed episodes of CRSV.

“Groups of men,” members of terrorist gangs, “men,” “rebels,” police officers, camp staff, Asma Boys, “boys gangs,” *madames*, smugglers, “armed militia” men, and militia men were identified as perpetrators of CRSV.

“The first time I attempted to come to Italy by boat I was arrested by the Libyan police and brought to prison. […] At night, policemen came and raped us. Men were beaten and we were also beaten if we refused to have intercourse with policemen.” (Nigeria)

CRSV was perpetrated indoors (e.g., the victim’s or other acquaintances or family members’ home), outdoors (e.g., street), as well as in enclosed or secluded spaces (e.g., concentration points, prisons, brothels, holding locations, camps, detention houses, on occasions generally defined as “houses” or “abandoned houses”).

"One day some Arab men came to our house and kidnapped us. They put us in a prison where there were many women […]. Some men arrived, took us away, raped us, and we couldn't fight back. This happened ceaselessly. I couldn't take it any longer, so I told them: "Just kill me, I am tired, I cannot do this anymore". But these men took me and dragged me on the floor […], hit me hard on my right arm, which still hurts to this day, and on my head. Sometimes I still feel dizzy because of this act of violence." (Ivory Coast)

Disclosed co-occurring acts of violence were blunt force injuries, ejection from a moving car, dragging, denial of care, witnessed death or violence on family members, death-related or other threats, housebreaking, theft, house destruction, detention, threat of guns, sharp force injuries, scourging, denial of food, burning, torture, and extortion.

#### 3.5.4 Negative consequences of CRSV

Negative outcomes of CRSV are reported in Table 3.

**Table 3:** Table showing the health consequences of CRSV.

Type of consequences	*n* (Total *N* = 43)
**Physical consequences**	21
*Sexual and reproductive health issues*	
Pregnancy	6
Violence-induced miscarriage	3
Bleeding	2
Abdominal and pelvic pain	2
Genito-anal injuries	1
Dysmenorrhea	1
Persistent abdominal pain	1
Scars on other erogenous sites (e.g., breasts)	1
*Other*	
Limb pain	3
Scars	3
Loss of consciousness	2
Foot pain and swelling	1
Inability to walk	1
Physical debilitation	1
High fever	1
Bone fractures	1
Onycholysis and ungual bed infection	1
Wounds on eyes and legs	1
Migraine, dizziness, and headache	1
**Psychological consequences**	31
Fear	19
Crying	14
Sadness	11
Horror	7
Insomnia or difficulties sleeping	6
Overthinking and rethinking about violence endured	4
Anxiety	4
Helplessness, detachment, emotional numbness, and wish for their life to end	2
Feeling of unsafety	1
Worry for their children	1
Shock	1
Fear-induced arousal	1
Freezing	1
Powerlessness	1
Alert	1
Restlessness	1
Shame	1
Loss of appetite	1
PTSD	1
Desperation	1
Hopelessness	1
Trauma	1

### 3.6 Other violence

In addition to CRSV, other episodes of conflict and migration related nonsexual violence and GBV were described by patients.

#### 3.6.1 Conflict and migration related nonsexual violence

In many instances, other violence was connected to the conflict encountered in victims’ home country or in transit, as well as to their migration experience.

In their home country, survivors reported personal and property offenses against themselves and family members. A woman reported her son dying during migration:

“I fled together with my younger child and other people to Sudan. At the border, soldiers started to shoot us and I fell. My son violently hit his head and died of a hemorrhage.” (Eritrea)

Violence on the move and in a transit country was described by 22 survivors (51%) and included property and personal offenses against themselves and family members. Some survivors who disclosed working as housemaids, or were labor trafficked, reported a variety of mistreatments, including physical violence, not being paid, and being sent out to the streets.

Arrests were mostly reported as occurring through policemen breaking into a victim’s home. At times they were connected to racism, or to migration control in concentration points or on while trying to leave for Italy. Survivors disclosed being sent to detention or prison camps or centers and being moved between different locations.

In this context, many victims described forceful separation from family members, attempted forced separation from children, beatings, torture, exploitation and mistreatment, threats, and starvation. One reported undergoing cesarean delivery in prison and being told her baby was dead. Extortion and money lent by strangers used as a form of ransom were also described.

"Every day they asked for extortion money. If people didn't have money, they threw water on them and then connected them to an electric plug and poured melted plastic on them. They hit people with sticks and raped them." (Ivory Coast)

Smuggling of migrants was described especially for what concerns the last phases before departure from the Libyan coast, occasionally connected to paying ransoms. Survivors disclosed receiving death threats, including with guns, while being forced to get on a boat and witnessing the murder of those who refused.

"One night, some Arab men arrived and under the threat of weapons they told me I had to go out of that house [and follow them]. During the route I was feeling very cold. Then I heard the sound of sea waves. Still under the threat of weapons, they made us get out of the car and we found ourselves on the shore. There were many people. With the use of force, they pushed us on a rubber boat, I didn't know where I was going." (Nigeria)

One reported being forced on a rubber boat and being sent adrift with the engine not working, those trying to get down being shot. Another disclosed being left on the shore because she was laboring.

Violence in the host country (*n* = 5) was related in most cases to their vulnerable condition after migration, with some of them reporting becoming unhoused. Survivors became aware of threats or extortion targeting relatives when living in the host country or in transit.

#### 3.6.2 Other GBV

In addition to experiences of CRSV, survivors encountered other instances of GBV during their lifetime. We separated GBV during childhood (*n* = 2), NPV (*n* = 6), and relational GBV, encompassing domestic and intimate partner violence (*n* = 7).

Before CRSV, some patients described being sexually trafficked (*n* = 3) or prior episodes of sexual violence victimization (*n* = 4).

## 4 Discussion

This study analyzed the interview transcripts of victims of CRSV accessing care at the sexual violence relief center *Soccorso Violenza Sessuale* (SVS) in Sant’Anna Hospital in Turin, North-West Italy. The most reported countries of origin of migrants were Ivory Coast, Nigeria, and Cameroun. In this study, 25 patients described their home country as conflict-affected, while 31 reported encountering hostilities in Libya. CRSV was disclosed by 18 survivors as occurring in their home country, where a commonly described pattern was perpetrators breaking into victims’ homes and assaulting them. All those transiting through Libya (*n* = 31) were subjected to CRSV there. This is coherent with data addressing the high prevalence of violence endured by migrants transiting this country (Castagna et al., 2018; Reques et al., 2020). CRSV was experienced in both home and transit countries by 14% of patients. Among the different types of CRSV, rape was the most disclosed. Perpetrators were hard to identify and, in most cases, had some form of authority over survivors, confirming other studies’ findings (Reques et al., 2020; Tan and Kuschminder, 2022).

Almost half of the patients described physical consequences of CRSV, the most disclosed one being pregnancy, in agreement with other literature findings (Rubini et al., 2023; Svallfors et al., 2024), and 72% reported psychological outcomes, in particular fear, crying, and sadness. Feelings of detachment and avoidance as perceived by members of the research team during the thematic analysis may also be connected to the effect of multiple traumatic events on victims (Lijtmaer, 2022; Verelst et al., 2020). While the Biopsychosocial Model proposed by Engel also encompasses the social dimension of health and even though negative consequences in this sphere are highly represented in populations affected by CRSV (Rubini et al., 2023), no SVS patient described social consequences. This could be related to social outcomes not being considered as part of their general health status by survivors and consequently not being disclosed or could be connected to providers not asking direct questions concerning this dimension of health during the interview, limiting the depth of our findings.

Many survivors described different forms of violence, including conflict and CRSV, as a driver for leaving their home country. A smaller number of patients traced back the cause for migration to economic reasons.

The majority of migratory flows in the years 2012–2017 occurred on the Central Mediterranean Route, the world’s deadliest migratory journey (Malakooti, 2019; Missing migrants project, n.d.). Patients from SVS described partially or entirely their migration route, which for the majority coincides with the Mediterranean one, with Libya being the last transit country for 86% of them. There, as documented by survivors’ accounts, conflict intertwines with the system of control, detention, exploitation, extortion, and smuggling of migrants (Reques et al., 2020). In addition, individuals who could be eligible for international protection in other States are identified in Libya as “irregular migrants,” since no law on asylum has been implemented and entering illegally in the country is a criminal offense punishable with imprisonment (Mancini, 2018). This situation presents some similarities with the context of the Mexico-USA migration corridor, characterized by high levels of violence and danger, as well as by captivity in “safe houses” and the presence of smugglers (e.g., *coyotes*). Moreover, migrants passing through Mexico are considered “irregular” and can be subjected to deportation, even though conflicts, organized crime, and GBV often constitute their reason to migrate (Cleaveland and Kirsch, 2020; Infante et al., 2020). Punitive measures enforced against irregular migrants render them at higher risk of abuse (Soria-Escalante et al., 2022).

In the years 2017–2023 there was a drastic reduction in the number of accesses at SVS, which can partially be traced to the decrease in the amount of arrivals in Italy, influenced by a Memorandum of Understanding signed between Italy and Libya in 2017 in which migration is framed as a “security issue” and where Italy agreed to provide financial and technical support to the Libyan Coastal Guard in order to increase their maritime surveillance capacity (Memorandum of understanding on cooperation in the fields of development, the fight against illegal immigration, human trafficking and fuel smuggling and on reinforcing the security borders between the State of Libya and the Italian Republic, 2017; MSF, 2022). The figure dropped in 2018 and 2019 (e.g., from 119,369 arrivals in 2017 to 23,370 and 11,471, respectively, in 2018 and 2019; UNHCR, n.d.), reaching the lowest number of arrivals since 2012 (Malakooti, 2019). This also coincided with the increase in the number of migrants intercepted at sea by the Libyan Coastal Guard and transferred into detention facilities (IRC, 2021).

The findings of the present study align with the notion of “conflict-affected women on the move” conceptualized by Holvikivi and Reeves, highlighting the long-term impact of conflict (Holvikivi and Reeves, 2020). In fact, the hostilities often constituted the first of a series of events carrying a negative impact on survivors’ well-being (e.g., forced migration, violent death of family members, detention, and torture). This also aligns with the concept of “ultraviolence,” namely the great level and different types of brutality employed by a variety of stakeholders in distinct moments in space and time, encompassing the conflict-related nonsexual violence, as well as CRSV and nonsexual violence inflicted during sexual assault, together with violence experienced in its aftermath (Rubini et al., 2023).

In Italy, in recent years, new protocols and trainings have been implemented for the management of GBV cases (Linee guida nazionali per le Aziende sanitarie e le Aziende ospedaliere in tema di soccorso e assistenza socio-sanitaria alle donne vittime di violenza, 2017; Ministero della Salute, 2023; Presidenza del consiglio di ministri Dipartimento Pari Opportunità, 2021), including laws on triage, with the mandatory assignment of at least a “yellow code” or equivalent (e.g., urgent care needed; Linee guida nazionali per le Aziende sanitarie e le Aziende ospedaliere in tema di soccorso e assistenza socio-sanitaria alle donne vittime di violenza, 2017). However, operator- (e.g., awareness of GBV, personal beliefs, and training) and health system-dependent (e.g., limited resources to conduct clinical and forensic tasks) obstacles still exist (Cattaneo et al., 2024; Gino and Caenazzo, 2020; Mtaita et al., 2023; Progetto REVAMP 2018, Istituto Superiore di Sanità, 2018). In addition, only two centers situated in the North-West area provide specialist care for GBV survivors, with only one of them accessible to cisgender men victims of violence (Margherita et al., 2021; Policlinico Milano, n.d.). This figure echoes the limited number of specialist centers available for the management of GBV in Europe, highlighting a gap between the needs of survivors and health services availability and accessibility (Rajan et al., 2021). Taking into consideration that the Explanatory report of the Council of Europe Convention on Preventing and Combating Violence against Women and Domestic Violence (e.g., Istanbul Convention) recommends one specialist service to be available to every 200.000 inhabitants (Council of Europe, 2018, 2011a, 2011b), with a geographic spread guaranteeing their accessibility in cities and rural areas, this objective is far to be met. Of the 1,400 hospitals on the Italian territory, 615^1^ have an ED (Pronto soccorso in Italia: Agenas presenta i dati alla Camera, 2023), each providing care for a pool of users of at least 80.000–150.000 inhabitants. This means that, according to the Explanatory report, roughly every ED in Italy should host a specialist service for the management of GBV cases. However, it would be more realistic for a smaller number of these centers to be present either at regional level or to cover large territorial areas.

The creation of intrahospital and centralized specialist services could reduce the pressure on general as well as on obstetrics and gynecological EDs, which constitute the main entry point for survivors of GBV (Ministero della Salute, 2023; Progetto REVAMP 2018, Istituto Superiore di Sanità, 2018; Regione Lombardia, 2021), including SV, alleviating the burden on the health system and on providers, who need to take care of patients presenting with a variety of health issues necessitating emergency and urgent care (Regione Lombardia, 2021). Implementing specialist services could ultimately result in better health outcomes and higher quality of care for survivors (Morley et al., 2018), also in terms of dedicated spaces within hospitals as well as longer consultation time for complex cases. In the meantime, better training for forensic and clinical management of patients accessing care due to GBV should be implemented in EDs (Cattaneo et al., 2024). This should also encompass continuous training for health providers on laws regulating the management of victims of GBV, including the legal requirements for reporting, also in cases of undisclosed abuse (Albert Henry, 2023).

As highlighted in the gatekeeping model proposed by Rajan et al. (2021), barriers in healthcare-seeking in cases of sexual abuse encompass external elements related to the healthcare systems as well as internal ones, connected to the patient (Rajan et al., 2021). The latter include difficulties in understanding the correlation between symptoms and violence, as well as shame, fear, and the effects of trauma on brain functions and abilities (Rajan et al., 2021). For migrant survivors, these difficulties intersect with other levels of obstacles in accessing care connected to language barriers (Trentin et al., 2023) and navigating the new health system in the host country (Pandey et al., 2022), as well as lack of confidence in the legal and healthcare systems (Rodella Sapia et al., 2020). For these reasons, it is plausible that the number of migrant women who need to access GBV-related care is larger compared to the current pool of users of specialist and general services within this population. Moreover, since continuity of care is hindered during the migration process, migrant women may suffer from health conditions exacerbated by delay in access to care (UNHCR, n.d.).

Given the high number of migrants arriving in Italy who encountered conflict and possibly CRSV, and taking into consideration that this population is among those at higher risk of GBV victimization (Progetto REVAMP 2018, Istituto Superiore di Sanità, 2018; Rodella Sapia et al., 2020), implementing specialist services for the management of GBV could also serve their complex health needs (Rubini et al., 2024) and guarantee the high quality of forensic practice that is needed to document the abuses they endured, possibly influencing the determination of protection outcomes (Rubini et al., 2024). They should also receive support in navigating the health system (Presidenza del consiglio di ministri Dipartimento Pari Opportunità, 2021), including GBV-related services, through dissemination of informative materials (e.g., in shops, reception venues, counseling centers), with language- and cultural-specific communication campaigns on the use of the National Helpline “Violence and Stalking” 1522 (Presidenza del consiglio di ministri Dipartimento Pari Opportunità, 2021), and the stable presence of cultural mediators in health facilities (Presidenza del consiglio di ministri Dipartimento Pari Opportunità, 2021). Culturally sensitive and migrant inclusive policies and protocols should be developed and implemented in all EDs. Migrants should also be aware of their entitlement to primary healthcare (Acquadro-Pacera, 2024).

Systems caring for migrants and anti-trafficking networks should act as a contact point between individuals seeking international protection belonging to the category of vulnerable persons, including victims of trafficking, and GBV-related services (Disposizioni urgenti in materia di flussi di ingresso legale dei lavoratori stranieri e di prevenzione e contrasto all’immigrazione irregolare, 2023; Ministero della Salute, 2017). These initiatives enabling access to care should equally target undocumented migrants who are victims of GBV, prioritizing their health needs and human rights over migration control (PICUM, 2024). The best practices implemented by SVS at Sant’Anna hospital in Turin, including fostering the connection with NGOs and other third sector organizations working with migrants, as well as the culturally sensitive approach to GBV, together with the positive results obtained through these practices in terms of equity in access to care, highlight the necessity of creating similar services in Italy in order to offer high quality care to migrants survivors of GBV, including CRSV. It is therefore crucial to integrate the systems taking care of migrant women with those managing the sequelae of GBV.

Implementing these recommendations could contribute towards the achievement of the United Nations’ 2030 SDGs, in particular number three—Healthy Lives and Well-Being, number five—Gender Equality, and number 16—Peaceful and Inclusive Societies (UN, 2015).

The study findings carry some limitations, as physical and psychological consequences refer to those described or manifested by patients during the interview and do not encompass those emerging through top-to-toe and gynecological examination. Moreover, they reflect the experiences of the pool of users of SVS and may not be generalizable.

## 5 Conclusion

This paper analyzed the history of violence as presented by cisgender women survivors of CRSV accessing care at the specialist center SVS in Sant’Anna hospital in Turin. The study confirmed the high level and different modalities of violence, including CRSV, experienced by patients in their home country as well as during their migratory journey, in particular in Libya. Many patients described being raped. More reported psychological outcomes were fear, crying, and sadness, while the most disclosed physical consequence was pregnancy. Qualitative analysis of interview transcripts was a valuable source for understanding how survivors described the CRSV they endured, its consequences, as well as other violence encountered during their migratory route. More studies should employ this methodology, also exploring the experiences of other populations affected by CRSV.

## Data Availability

The data analyzed in this study is subject to the following licenses/restrictions: the datasets used and/or analyzed during the current study are available from the corresponding author on reasonable request. Requests to access these datasets should be directed to Elena Rubini, elena.rubini@uniupo.it.

## Footnotes

^1^This figure represents the total number of hospitals with an ED, including secondary- and tertiary- level hospitals.
